# Studies towards the synthesis of hyperireflexolide A

**DOI:** 10.3762/bjoc.14.185

**Published:** 2018-08-13

**Authors:** G Hari Mangeswara Rao

**Affiliations:** 1 Department of Chemistry, Indian Institute of Technology Kanpur, Kanpur-208016, India; 2 Department of Chemistry, Texila American University, Georgetown, Guyana

**Keywords:** alkylation, allylation, cross metathesis, hyperireflexolide A, spiroterpene

## Abstract

The first approach to hyperireflexolide A, based on the synthesis of γ-lactone-fused cyclopentane **5**, a functionalized key intermediate, is presented. Compound **5** is involved in hydrolysis, α-allylation at C-8 and α-methylation at C-10 stereoselectively from the convex face. Then it is subjected to cross metathesis to give α*,*β-unsaturated ketone **11** as precursor in the total synthesis of hyperireflexolide A.

## Introduction

**Figure 1:**
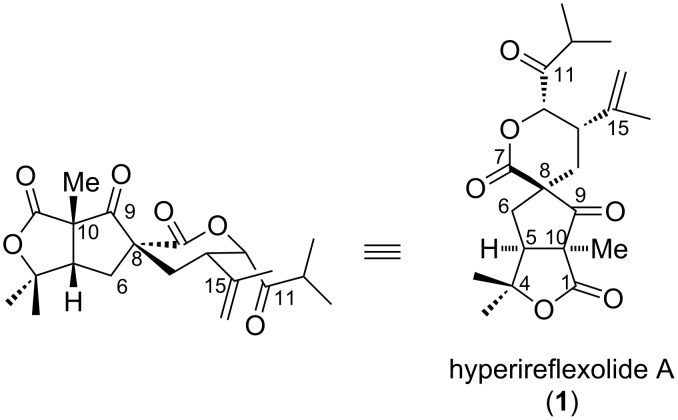
Hyperireflexolide A.

Hyperireflexolide A (**1**) [1] is a spiroterpenoid, isolated from *hypericum reflexum,* plants of the genus hypericum (Figure 1). Hyperireflexolide A is widely used in folk medicine, displays antifungal [2] and cytotoxic activities [3].

γ-Lactone-fused cyclopentanes are of vital importance in organic synthesis and are the most abundant substructures found in various naturally occurring molecules [4–5]. A *cis*-cyclopentane ring-fused γ-lactone is the key structural unit of many complex and challenging biologically active natural products [6–19]. The γ-lactone-fused cyclopentane ring system is also an important component for the synthesis of a variety of cyclopentanoid natural and unnatural products [20–24].

In the literature, numerous synthetic methods are reported to attain γ-lactone-fused cyclopentanes [25–31]. Earlier from our lab, we reported a short and efficient methodology for the synthesis of γ-lactone-fused cyclopentane **5** [32]. The *cis*-ring junction of this carbocylic ring system offers a high degree of selectivity for the assemblage of various substituents on the convex surface. The lactone part can serve as a useful tool to append various side chains.

## Results and Discussion

The presence of a γ-lactone-fused cyclopentane moiety in hyperireflexolide A (**1**) attracted our attention. In fact, the ketal moiety in **5** could not only act as a surrogate for C-9 carbonyl but also facilitate installation of an angular methyl group.

**Scheme 1:**
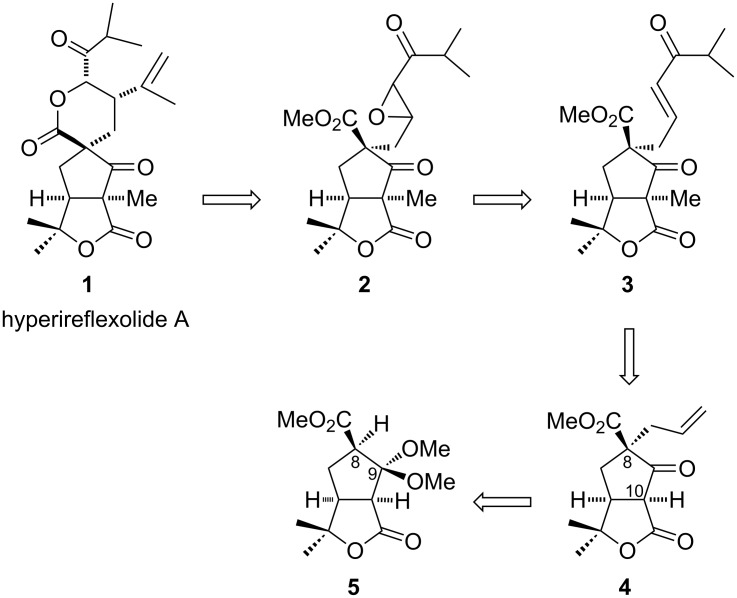
Retrosynthetic strategy.

The retrosynthetic analysis for hyperireflexolide A is depicted in Scheme 1. We envisioned that hyperireflexolide A (**1**) could be synthesized by metal-catalyzed opening of the epoxide **2** with 2-bromopropene followed by lactonization. Enone **3** could be synthesized from **4** by installation of the methyl group at C-10 followed by cross metathesis reaction. Compound **4** could be obtained from the γ-lactone-fused cyclopentane **5** by deprotection of C-9 followed by allylation at C-8.

**Scheme 2:**
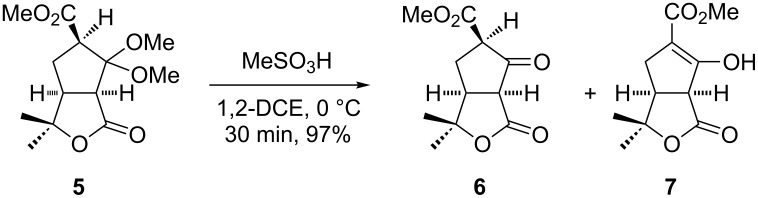
Hydrolysis of dimethyl ketal **5**.

Previously, we reported from our laboratory the synthesis of γ-lactone-fused cyclopentane derivative **5** from the respective Diels–Alder adduct in 5 steps with an overall yield of 29% [32]. Hydrolysis of dimethyl ketal **5** with MeSO_3_H in 1,2-DCE furnished γ-lactone-fused cyclopentanone **6** in 97% yield. Cyclopentanone **6** exists in its tautomeric enol form **7**, observed in the ^1^H NMR spectrum after column chromatographic purification (**6** and **7** were not separated) as represented in Scheme 2 [33].

**Scheme 3:**
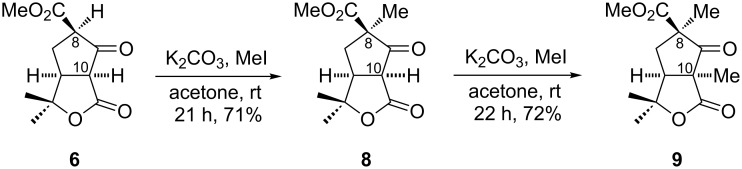
Alkylation of γ-lactone-fused β-ketoester **6**.

In order to check the feasibility of the alkylation reaction of γ-lactone-fused β-ketoester **6**, initially a mixture of **6** and **7** was subjected to methylation using 1.1 equiv of K_2_CO_3_ in the presence of methyl iodide (MeI). The α-methylated β-ketoester **8** was obtained in good yield. In the ^1^H NMR, **8** showed a signal at 3.70 ppm as doublet for C-10 (ring junction) proton confirming the selective methylation at C-8. Further, installation of the methyl group at C-10 was achieved by treatment of **8** with 1.1 equiv of K_2_CO_3_ in the presence of MeI to give bis-methylated γ-lactone-fused β-ketoester **9** in 72% yield (Scheme 3). These results demonstrated that regioselective alkylation at the two sites were possible. Notably, one diastereomeric product was isolated from these bis-alkylation reactions due to favorable attack from the less hindered convex face of **6** [10] to give α,α'-*cis* stereochemistry.

**Scheme 4:**
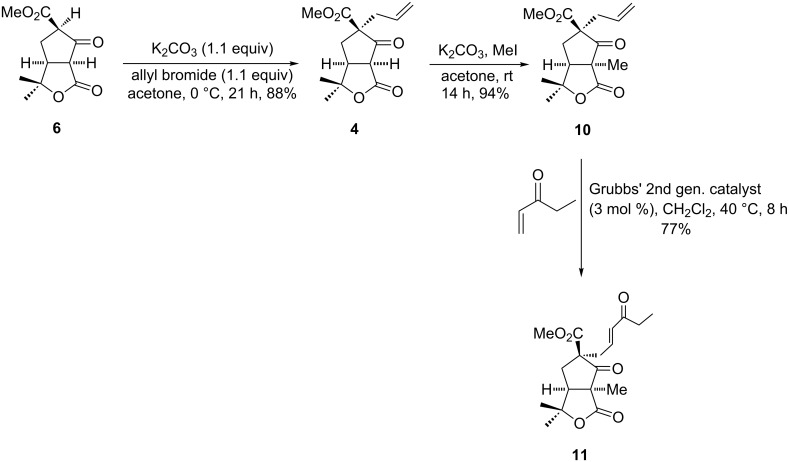
Synthesis of α*,*β-unsaturated ketone **11**.

Now the stage has been set for allylation of γ-lactone fused cyclopentanone **6**. Treatment of **6** with K_2_CO_3_ in the presence of allyl bromide at 0 °C afforded α-allylated γ-lactone-fused β-ketoester **4** in 88% yield. In the ^1^H NMR a signal appeared at 3.64 ppm as doublet for ring junction (C-10) proton confirmed that selective allylation occurred at C-8. Compound **4** was then subjected to methylation at C-10 using K_2_CO_3_ and MeI to obtain the requisite γ-lactone fused cyclopentanone **10** in excellent yield (Scheme 4). Allylation and methylation both were occurred stereoselectively from the convex face to give α,α'-*cis* stereochemistry. The allyl derivative **10** was then subjected to cross metathesis reaction with ethyl vinyl ketone. Initially, the reaction performed using Grubbs’ 1st generation catalyst (3–20 mol %) was unsuccessful. Treatment of **10** with ethyl vinyl ketone using Grubbs’ 2nd generation catalyst (3 mol %) in the presence of CH_2_Cl_2_ furnished (*E*)-enone **11** in 77% yield as shown in Scheme 4. In the ^1^H NMR spectrum, **11** showed a signal at 6.13 ppm as doublet with coupling constant 15.8 Hz (*trans*-configuration) for the α-proton of enone [34–37].

After successfully synthesizing the side chain via cross-metathesis, our next task was the steresoselective epoxidation of (*E*)-enone **11**. Unfortunately, the stereoselective epoxidation of **11** under basic conditions were unsuccessful [38], which prevented completion of the proposed synthetic sequence.

## Conclusion

In conclusion, synthetic studies towards hyperireflexolide A, the synthetic precursor α*,*β-unsaturated ketone **11** was synthesized. Failure of the stereoselective epoxidation of **11** prevented completion of the proposed synthetic sequence. Future studies will include the stereoselective epoxidation of **11** followed by opening of the epoxide and lactonization or 1,4-nucleophilic addition to the α*,*β-unsaturated ketone **11** followed by epoxidation of the resulted enolate with subsequent lactonization to achieve hyperireflexolide A (**1**).

## Experimental

### General methods

All the reactions were performed in oven dried apparatus and the reaction mixtures were magnetically stirred. Thin-layer chromatography was performed on Acme and Spectrochem Silica gel (Mumbai, India) coated on microscopic slides. Visualization of spots was effected by exposure to iodine or spraying with 4% ethanolic H_2_SO_4_ and charring. Column chromatography was performed using Acme's silica gel (100–200 mesh), and ethyl acetate/hexane was used as eluent. Evaporation of solvents was performed at reduced pressure using a Büchi rotary evaporator.

Melting points were recorded on JSGW melting point apparatus and are uncorrected. Infrared spectra were recorded on Perkin-Elmer 1320 and Shimadzu 420 spectrophotometers as KBr pellets (solids), or as thin films on NaCl flats (liquids). ^1^H NMR spectra were recorded at 400 MHz on a JEOL spectrometer unless otherwise mentioned (500 MHz). Data are reported as follows: (br = broad, s = singlet, d = doublet, t = triplet, q = quartet, m = multiplet; integration; coupling constant(s) in Hz; assignment). Chemical shifts are reported in ppm, and coupling constants are reported in Hz. Proton decoupled ^13^C NMR spectra were recorded at 100 MHz (125 MHz) on a JEOL spectrometer. Samples for NMR were made in CDCl_3_. Tetramethylsilane was used as the internal standard.

Commercial grade solvents were distilled before use. Ethyl acetate was distilled over anhydrous sodium carbonate. Dichloromethane and dichloroethane (1,2-DCE) were distilled over phosphorous pentoxide and stored over 4 Å molecular sieves. Acetone was distilled over anhydrous K_2_CO_3_. Methanol was refluxed and distilled over magnesium turnings and stored over 4 Å molecular sieves. Distilled water was used for aqueous reactions and aqueous work-up.

### Experimental procedures and analytical data

**Methyl 1,1,5-trimethyl-3,4-dioxohexahydro-1*H*-cyclopenta[*c*]furan-5-carboxylate (8):** To a solution of the γ-lactone-fused cyclopentanone **6** (16 mg, 0.07 mmol) in dry acetone (0.4 mL) under argon was added anhydrous K_2_CO_3_ (9.7 mg, 0.07 mmol) and methyl iodide (12 mg, 0.08 mmol) at 0 °C. The reaction was stirred at room temperature for 21 h. Completion of the reaction was monitored by tlc. The reaction was diluted with water (3 mL) and the organic layer was extracted with EtOAc (3 × 3 mL). The combined organic layers were washed with brine solution (2 mL) and dried over anhydrous Na_2_SO_4_. The solvent was concentrated in vacuo to furnish a residue which was purified by silica gel column chromatography (25% EtOAc/hexane) to afford **8** in 71% yield. Viscous liquid; ^1^H NMR (400 MHz, CDCl_3_) δ 3.72 (s, 3H, CO_2_Me), 3.70 (d, *J* = 1.9 Hz, 1H), 2.98–2.91 (m, 1H), 2.52 (dd, *J* = 13.2, 11.2 Hz, 1H), 2.11–2.04 (m, 1H), 1.50 (s, 3H, Me), 1.47 (s, 3H, Me), 1.37 (s, 3H, Me); ^13^C NMR (100 MHz, CDCl_3_) δ 203.4 (C=O), 171.0, 167.7, 84.5, 58.0, 55.6, 52.8, 44.9, 35.1, 29.3, 22.3, 18.7; IR (neat): 2900, 1760, 1740, 1440 cm^−1^; HRMS (*m*/*z*): [M]^+^ calcd for C_12_H_16_O_5_, 240.0998; found, 240.1001.

**Methyl 1,1,3a,5-tetramethyl-3,4-dioxohexahydro-1*H*-cyclopenta[*c*]furan-5-carboxylate (9):** To a solution of the γ-lactone-fused cyclopentanone **8** (15 mg, 0.07 mmol) in dry acetone (0.4 mL) under argon was added anhydrous K_2_CO_3_ (9.7 mg, 0.07 mmol) and methyl iodide (12 mg, 0.08 mmol) at 0 °C. The reaction was stirred at room temperature for 22 h. Completion of the reaction was monitored by tlc. The reaction was diluted with water (3 mL) and the organic layer was extracted with EtOAc (3 × 3 mL). The combined organic layers were washed with brine solution (2 mL) and dried over anhydrous Na_2_SO_4_. The solvent was concentrated in vacuo to furnish a residue which was purified by silica gel column chromatography (20% EtOAc/hexane) to afford **9** in 72% yield. ^1^H NMR (400 MHz, CDCl_3_) δ 3.70 (s, 3H, CO_2_Me), 2.66 (t, *J* = 8.6 Hz, 1H), 2.55 (dd, *J* = 13.9, 8.8 Hz, 1H), 2.05 (dd, *J* = 13.9, 8.4 Hz, 1H), 1.64 (s, 3H, Me), 1.60 (s, 3H, Me), 1.49 (s, 3H, Me), 1.36 (s, 3H, Me); ^13^C NMR (100 MHz, CDCl_3_) δ 201.4 (C=O), 169.3, 168.4, 80.2, 59.2, 56.4, 53.5, 44.4, 36.5, 28.7, 22.6, 19.8, 18.7; IR (neat): 2900, 1760, 1740, 1440 cm^−1^; HRMS (*m*/*z*): [M]^+^ calcd for C_13_H_18_O_5_, 254.1154; found, 254.1155.

**Methyl 5-allyl-1,1-dimethyl-3,4-dioxohexahydro-1*H*-cyclopenta[*c*]furan-5-carboxylate (4):** To a solution of the γ-lactone-fused cyclopentanone **6** (75 mg, 0.331 mmol) in dry acetone (1 mL) under argon was added anhydrous K_2_CO_3_ (55 mg, 0.3972 mmol) and allyl bromide (48 mg, 0.3972 mmol) at 0 °C. The reaction was stirred at 0 °C for 21 h. Completion of the reaction was monitored by tlc. The reaction was diluted with water (4 mL) and the organic layer was extracted with EtOAc (3 × 5 mL). The combined organic layers were washed with brine solution (3 mL) and dried over anhydrous Na_2_SO_4_. The solvent was concentrated in vacuo to furnish a residue which was purified by silica gel column chromatography (15% EtOAc/hexane) to afford **4** in 88% yield. Viscous liquid; ^1^H NMR (500 MHz, CDCl_3_) δ 5.72–5.64 (m, 1H), 5.18–5.12 (m, 2H), 3.71 (s, 3H, CO_2_Me), 3.64 (d, *J* = 7.2 Hz, 1H), 2.86–2.80 (m, 1H), 2.70 (dd, *J* = 13.8, 6.5 Hz, 1H), 2.50–2.45 (m, 1H), 2.35–2.30 (m, 1H), 2.25–2.20 (m, 1H), 1.50 (s, 3H, Me), 1.44 (s, 3H, Me); ^13^C NMR (125 MHz, CDCl_3_) δ 202.6, 169.9, 167.8, 131.8, 120.2, 84.8, 61.8, 56.2, 53.0, 44.5, 37.3, 31.2, 29.4, 22.3; IR (neat): 2900, 1760, 1720, 1420 cm^−1^; HRMS (*m*/*z*): [M]^+^ calcd for C_14_H_18_O_5_, 266.1154; found, 266.1150.

**Methyl 5-allyl-1,1,3a-trimethyl-3,4-dioxohexahydro-1*H*-cyclopenta[*c*]furan-5-carboxylate (10):** To a solution of the γ-lactone-fused cyclopentanone **4** (29 mg, 0.109 mmol) in dry acetone (0.6 mL) under argon was added anhydrous K_2_CO_3_ (16.5 mg, 0.1199 mmol) and methyl iodide (18.5 mg, 0.1308 mmol) at 0 °C. The reaction was stirred at room temperature for 14 h. Completion of the starting material was monitored by tlc. The reaction was diluted with water (3 mL) and the organic layer was extracted with EtOAc (3 × 4 mL). The combined organic layers were washed with brine solution (3 mL) and dried over anhydrous Na_2_SO_4_. The solvent was concentrated in vacuo to furnish a residue which was purified by silica gel column chromatography (10% EtOAc/hexane) to afford **10** in 94% yield. Viscous liquid; ^1^H NMR (500 MHz, CDCl_3_) δ 5.69–5.61 (m, 1H), 5.17–5.11 (m, 2H), 3.69 (s, 3H, OMe), 2.66 (dd, *J* = 13.9, 6.5 Hz, 1H), 2.57 (t, *J* = 8.5 Hz, 1H), 2.49 (dd, *J* = 14.0, 8.5 Hz, 1H), 2.35 (dd, *J* = 13.9, 7.5 Hz, 1H), 2.19 (dd, *J* = 14.0, 8.5 Hz, 1H), 1.59 (s, 3H, Me), 1.53 (s, 3H, Me), 1.50 (s, 3H, Me); ^13^C NMR (100 MHz, CDCl_3_) δ 206.0, 171.8, 170.3, 131.9, 120.1, 84.0, 60.7, 60.1, 52.8, 50.4, 37.8, 30.5, 30.2, 23.7, 22.5; IR (neat): 2900, 1760, 1740, 1460 cm^−1^; HRMS (*m*/*z*): [M]^+^ calcd for C_15_H_20_O_5_, 280.1311; found, 280.1310.

**Methyl 1,1,3a-trimethyl-3,4-dioxo-5-((*E*)-4-oxohex-2-enyl)hexahydro-1*H*-cyclopenta[*c*]furan-5-carboxylate (11):** To a solution of the compound **10** (110 mg, 0.327 mmol) in CH_2_Cl_2_ (0.8 mL) was added ethyl vinyl ketone (31 mg, 0.3597 mmol) and 3 mol % Grubbs’ 2nd generation catalyst (8.5 mg, 0.00981 mmol), then heated the reaction mixture at 40 °C for 8 h (monitored by tlc). The solvent was concentrated in vacuo to furnish a residue which was purified by silica gel column chromatography (15% EtOAc/hexane) to afford **11** in 77% yield. Viscous liquid; ^1^H NMR (500 MHz, CDCl_3_) δ 6.67–6.61 (m, 1H), 6.13 (d, *J* = 15.8 Hz, 1H, -C(O)-CH=C-), 3.70 (s, 3H, CO_2_Me), 2.80–2.75 (m, 1H), 2.59–2.51 (m, 4H), 2.48–2.43 (m, 1H), 2.12 (dd, *J* = 13.1, 7.4 Hz, 1H), 1.58 (s, 3H, Me), 1.53 (s, 3H, Me), 1.49 (s, 3H, Me), 1.07 (t, *J* = 7.4 Hz, 3H); ^13^C NMR (125 MHz, CDCl_3_) δ 205.6, 200.3, 171.5, 169.9, 139.1, 134.0, 84.1, 60.3, 60.1, 53.1, 50.5, 36.1, 33.5, 31.0, 30.3, 23.7, 22.5, 7.8; IR (neat): 2900, 1760, 1720, 1670, 1620, 1420 cm^−1^; HRMS (*m*/*z*): [M]^+^ calcd for C_18_H_24_O_6_, 336.1573; found, 336.1576.

## Supporting Information

File 1 Copies of ^1^H and ^13^C NMR spectra.
