# Supplementary material for: Studies towards the synthesis of hyperireflexolide A
Source: Beilstein J Org Chem. 2018 Aug 13;14:2106–11. doi: 10.3762/bjoc.14.185 (PMC6122383; doi:10.3762/bjoc.14.185)

Supporting Information

for

Studies towards the synthesis of hyperireflexolide A

G. Hari Mangeswara Rao[*,1,2]

Address: [1]Department of Chemistry, Indian Institute of Technology Kanpur, Kanpur-208016, India and [2]Department of Chemistry, Texila American University, Georgetown, Guyana

Email: G. Hari Mangeswara Rao[1] - harimangesh@gmail.com

*Corresponding author

Copies of ^{1}H and ^{13}C NMR Spectra

^{1}H and ^{13}C NMR Spectra

^{1}H NMR (500 MHz) and ^{13}C NMR (125 MHz) of **4**

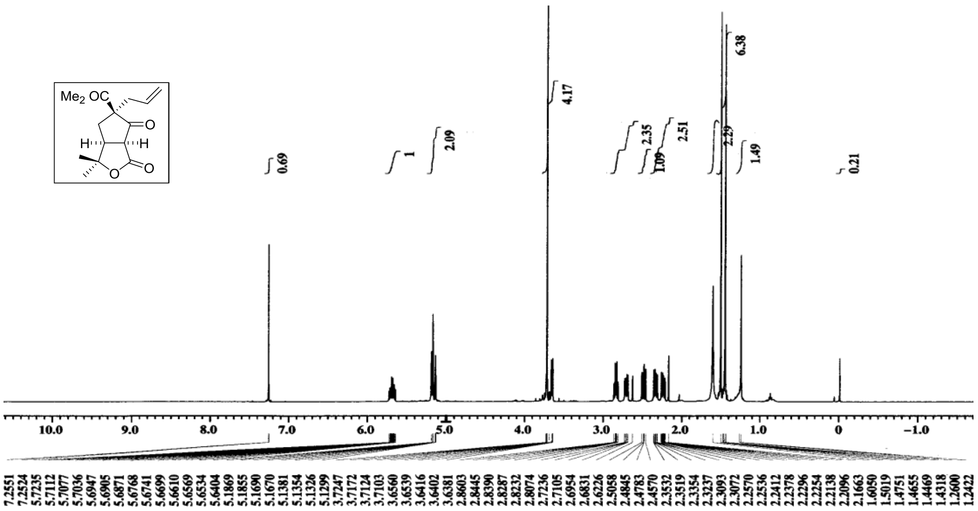

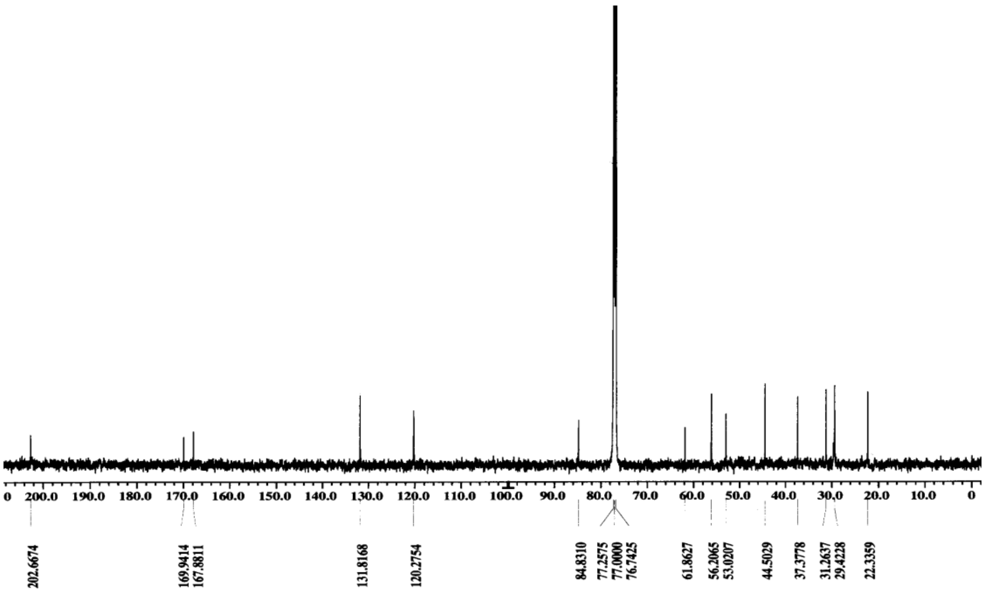

^{1}H NMR (500 MHz) and ^{13}C NMR (100 MHz) of **10**

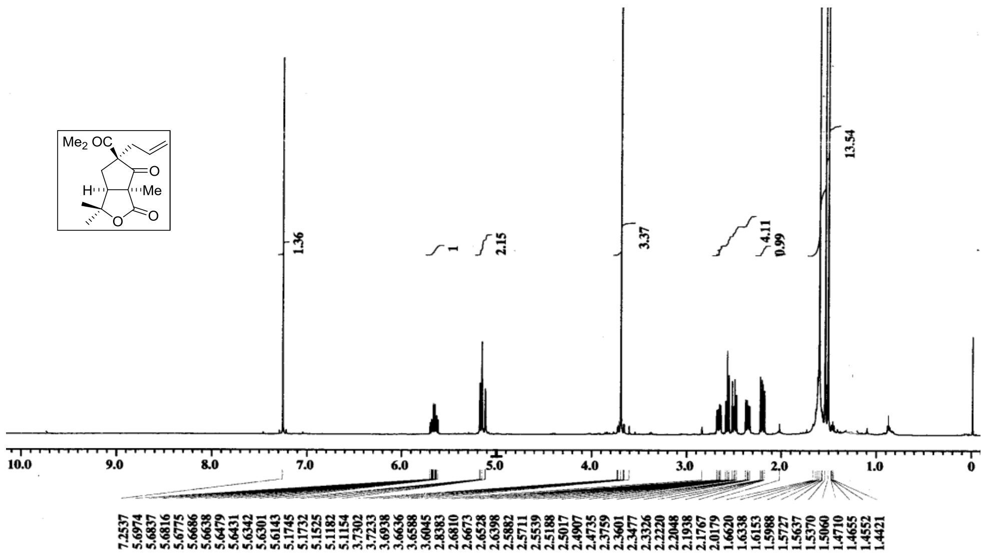

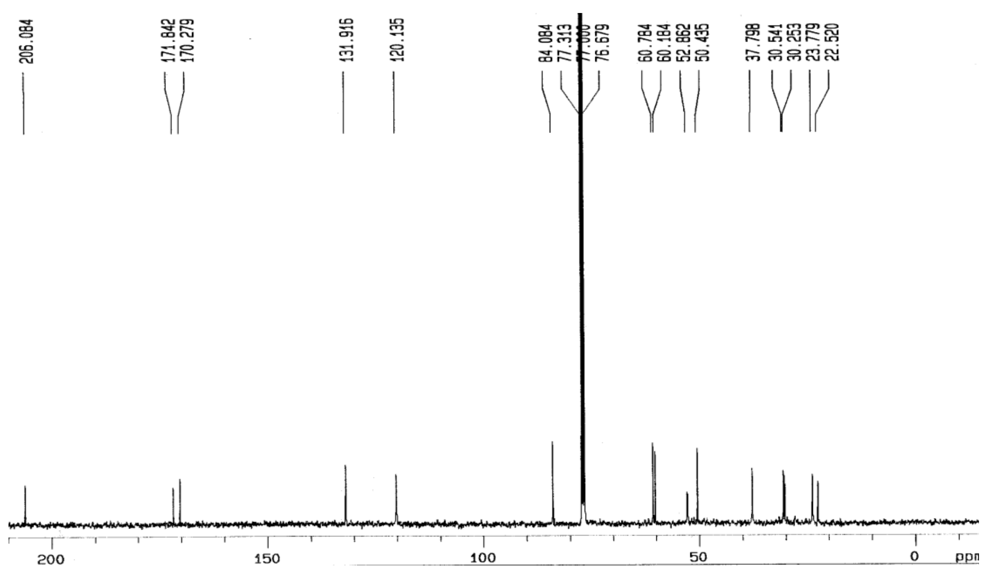

^{1}H NMR (500 MHz) and ^{13}C NMR (125 MHz) of **11**

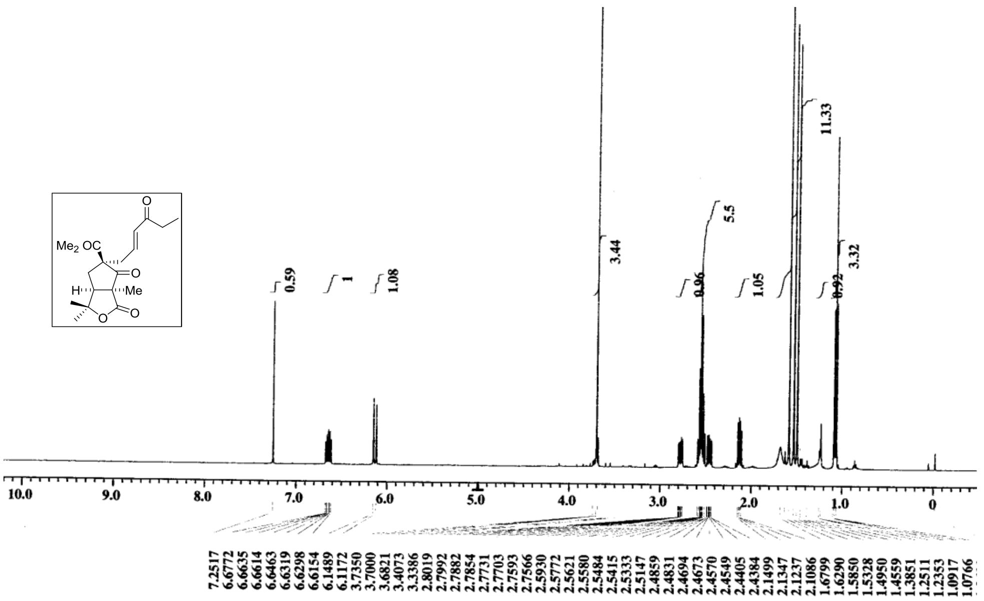

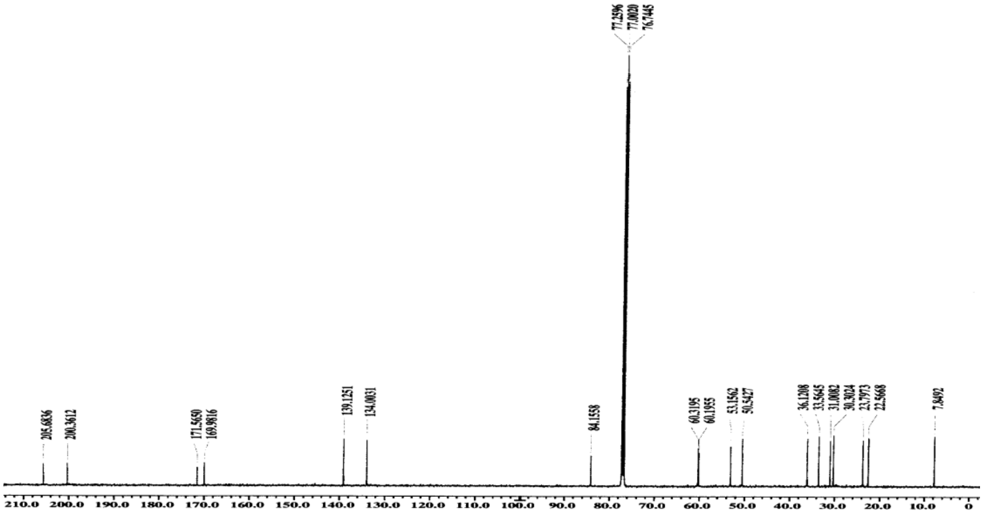

Supplement: File 1 — Copies of 1H and 13C NMR spectra. [file Beilstein_J_Org_Chem-14-2106-s001.pdf]
